# Synthesis and Appraisal of Natural Drug-Polymer-Based Matrices Relevant to the Application of Drug-Eluting Coronary Stent Coatings

**DOI:** 10.1155/2020/4073091

**Published:** 2020-11-17

**Authors:** Bakhtawar Ghafoor, Murtaza Najabat Ali, Zainab Riaz

**Affiliations:** Biomedical Engineering & Sciences Department, School of Mechanical and Manufacturing Engineering, National University of Sciences and Technology (NUST), Sector H-12, Islamabad, Pakistan

## Abstract

Cardiovascular diseases are becoming a leading cause of death in the world, and attention is being paid to develop natural drug-based treatment to cure heart diseases. Curcumin, ginger, and magnolol are pharmaceutically active in many ways, having properties including anticoagulation, antiproliferation, anti-inflammatory, and antioxidant, and may be used to synthesis coatings for drug-eluting stents to treat cardiovascular diseases. In the present investigation, a degradable polymer with varying molecular weights was used as a drug carrier to control the degradation of polymer; three different natural drugs such as curcumin, magnolol, and ginger were used owing to their reported pharmacological properties. The results of in vitro measurements of all three natural drugs released from drug-loaded polymeric films showed an initial burst release followed by a sustained release for up to 38 days of measurement. On the other hand, different levels of hemocompatibility were observed by varying concentrations of natural drugs in human erythrocytes. As per the ASTM F756 standard, ginger having low concentration showed optimum hemocompatibility with regard to the drug-eluting stent application as compared with magnolol and curcumin concentrations, which showed suboptimal hemocompatibility and fall in the range of mild-to-severe blood toxicity category. The structure of the coating films was characterized by Fourier transform infrared (FTIR) spectroscopy and scanning electron microscopy (SEM) with results suggesting that there was no chemical bonding between the polymer and drug. Thus, according to this study, it can be concluded that after more detailed in vitro testing such as hemocompatibility tests and platelet adhesion testing, ginger can be a better candidate as a drug-coating material for drug-eluting stent applications.

## 1. Introduction

Every year, the ratio of deaths due to cardiovascular diseases is increasing dramatically [1]. The primary cause of coronary vascular diseases (CVDs) is the development of atherosclerotic lesions in the arterial wall obstructing blood flow to the heart tissues by narrowing the arterial lumen. This condition is known as atherosclerosis. The underlying source of atherosclerosis is the high cholesterol levels in blood, which along with other factors such as smoking and hypertension causes dysfunctioning of the endothelium (the inner most layer of artery). Low-density lipids (LDLs) taking advantage of endothelium malfunctioning enter into the intimal layer, initiating the cascade of immune response. The presence of LDLs in the intima causes the influx of macrophages that engulf the LDLs and become foam cells, the hallmark of atherosclerosis. The formation of foam cells and release of growth factors lead towards the migration and proliferation of smooth muscle cells (SMCs) from the medial layer to intima. SMC proliferation results in the formation of the fibrous cap containing a lipid-rich necrotic core with extracellular matrix (ECM) depositions, and this fibrous cap is known as plaque. The plaque also known as atheroma hinders the blood flow, which can lead to severe cardiac arrest and may also end up in death of the patient [2–4].

Although significant improvement has been made in the treatment of patients suffering from coronary artery disease with the introduction of coronary stents, neointimal proliferation leading to in-stent restenosis remained a challenge. The arterial recoil and remodeling, thrombus formation, and vascular smooth muscle cell (VSMC) proliferation cause neointimal hyperplasia and intracellular matrix synthesis. These processes, the source of lumen thickening, are the major events that lead towards in-stent restenosis after stent implantation [5, 6]. Thus, attention was devoted towards treatment options that will target VSMC proliferation.

The drug-eluting stent (DES) can provide luminal scaffolding to eliminate arterial recoil, cellular proliferation, and thrombus formation by releasing antiproliferative drugs from the polymeric matrix. It is, therefore, desirable to develop the DES not only with a biocompatible and biodegradable coating to prevent these unfavorable effects but also loaded with effective drugs that either promote re-endothelization or suppress inflammation and VSMC proliferation [7]. Polyvinyl alcohol (PVA) has been in use as a drug carrier for drug delivery systems for many years because it is biocompatible, biodegradable, hydrophilic in nature with excellent film-forming property, flexibility, and good mechanical strength and is cost-effective and readily available [8]. In addition to that the presence of many polar groups and high chain flexibility make it a better option because these properties help in drug binding and control release of drugs in an aqueous environment when used for biomedical applications [9, 10].

The practice of using folk medicinal plants as traditional medicines has very deep roots in human history [11]. The pharmacologically active compounds found in plants have properties such as antioxidant, anti-inflammatory, anticancerous, and antimicrobial. Thus, before using these plants in the field of medicine such as coatings for medical implants, their cytotoxicity and biocompatibility needs to be evaluated. Previous studies have reported that many plants have hemolytic or antihemolytic effects on human erythrocytes [11–13].

Curcumin (diferuloyl methane), a major phytochemical component of turmeric, is extracted from the rhizome of *Curcuma longa*. Curcumin has a wide range of use in biomedical applications because of its antithrombotic, antioxidant, antiproliferative, anti-inflammatory, and anticarcinogenic properties [14–17]. On account of its vast pharmacological properties, curcumin is a potential candidate to be used for drug-eluting stents. Magnolol is a polyphenol found in *Magnolia officinalis*. It has been reported to have constituents that are beneficial for cardiovascular diseases. Magnolol inhibits intimal hyperplasia by inhibiting vascular smooth muscle cell proliferation and migration [18–20]. Additionally, it has antitumor, antimicrobial, anti-inflammatory, and antioxidant activities [21–23]. Ginger is a very old folk medicine widely used as a common home spice for about two centuries. Polyphenolic components such as 6-gingerol and its derivatives are present in its extract, which too have many pharmacological properties like antitumorigenic, anti-inflammatory, antiapoptotic, and immunomodulatory [24, 25].

In the present contribution, the synthesis of three natural drug-loaded polymeric films, i.e., using magnolol, curcumin, and ginger, was carried out using the solvent casting method. Polyvinyl alcohol (PVA) with varying molecular weights, in order to control the degradation rate of the polymer, is used as a drug carrier. The best candidate with respect to their hemolysis assay, in vitro drug release, and degradation studies was selected as a coating material for drug-eluting stents for cardiovascular diseases.

## 2. Materials

Three plant materials were recruited in this study based on their reported pharmacological properties such as inhibition of neointimal hyperplasia, antioxidant, and antiproliferation. The three selected plant extract-based drugs were magnolol purchased from Tauto (China), curcumin purchased from Alf Aesar (Germany), and ethanolic extract of ginger that was prepared and used in this research work. PVA with varying molecular weights, such as 72,000 KDa and 100,000 KDa, was selected based on its reported biodegradable and biocompatible properties. Dimethyl sulfoxide (DMSO) was used as a solvent because of its superior solubility with the plant extract.

### 2.1. Ethanolic Extraction of Ginger

The raw ginger was washed with clean water and allowed to air dry to reduce the microbial load of the plant material during handling and transportation. The outer covering of ginger was peeled off, and the material was sliced into cutlets. The material was placed in a hot air oven for drying and pulverized into powder using grinder.

The preparation of ethanolic extract of ginger was carried out by dissolving preweighed amount of ginger (0.025%) in 80% ethanol and the solution was stirred for 24 hrs. Subsequently, the mixture was filtered and the orange-yellow-colored supernatant was collected and evaporated at 80°C and stored in a covered Petri plate at 4°C for further use.

### 2.2. Film Fabrication

The preparation of drug-loaded polymeric films was carried out by dissolving PVA (3% low and high molecular weight) in DMSO through constant stirring for 1 hr. Subsequently, drugs (5%, 10%, and 15% w/v) were added into the PVA solution and the solution was stirred. The homogenous solution was poured into a Petri plate and dried at 37°C after 24 hrs. The compositions of the films are shown in Tables 1–3.

### 2.3. Degradation Studies

In vitro degradation of the drug-loaded membranes is represented as a function of weight loss over time at 37°C. The dried composite membranes were cut into pieces with a diameter of 1 cm × 1 cm. All preweighed specimens were immersed in capped vials containing 10 mL PBS (pH 7.4). The buffer solution was refreshed at a regular time interval. At predetermined time intervals, the samples were removed from the tubes and weighed (*W*_*t*_). The weight loss percentage (Δ*W*%) at each time interval was calculated using the following equation:

(1) $$\begin{array}{c} \Delta W\%=\frac{\left(W_{0}-W_{t}\right)}{W_{0}}*100, \end{array}$$

where *W*_0_ is the initial weight of each sample. All results were estimated from the data of three individual experiments, and the reported data were expressed as mean ± SD.

### 2.4. Drug Release Studies

The drug release profile was investigated in vitro by incubating film samples in a glass tube containing 3.0 mL medium at 37°C. The medium consisted of phosphate-buffered saline and in the case of curcumin containing 10% ethanol (v/v) [26] (because the limit of solubility of curcumin in water makes it impossible to study in buffer). At selected time periods, the incubation medium was completely removed for analysis and replaced with a fresh medium. An aliquot of the sampled medium was measured by UV-vis spectrophotometry at 284, 290, and 435 nm for magnolol, ginger, and curcumin, respectively. Results were expressed as cumulative micrograms and weight percent of curcumin released.

### 2.5. Hemolysis Assay

Blood compatibility of drug-loaded polymeric films was evaluated using hemolysis assay. PVA-based drug-loaded films were placed in PBS. Fresh human blood was obtained in a blood bag containing anticoagulants. For 2 mL of the whole blood sample, 4 mL phosphate-buffered saline (PBS) was added and centrifuged at 5000 rpm for 5 min to isolate red blood cells (RBCs). RBCs were further washed three times with PBS. The suspension of RBCs (2% v/v) was exposed to PBS suspension of drug-eluting polymeric films. The 0.5% of Triton X-100 was used as positive control and PBS as negative control. After incubation at room temperature for 4 h, cells were centrifuged for 5 min at 5500 rpm, the supernatant was collected, and the absorbance was measured using UV-vis spectrophotometry at 540 nm. Hemolysis percentage for each sample was calculated by dividing the sample's absorbance on positive control absorbance (complete hemolysis) and multiplied by 100 [27].

### 2.6. Scanning Electron Microscopy (SEM)

Scanning electron microscopy (SEM) was performed to find out the surface morphology of the casted films. The assessment of the surface morphology of the natural drug-based polymeric films was done using a JSM-6490A analytical scanning electron microscope (JEOL, Tokyo, Japan). SEM images were collected at an activation voltage of 20 kV. The polymeric drug-eluting blend films were placed on a metal sample holder and sputter coated (Emitech K-575 Sputter Coater) with gold-palladium at a thickness of 7.5 nm.

### 2.7. Fourier Transform Infrared (FTIR) Analysis

Fourier transform infrared (FTIR) spectroscopy (PerkinElmer; Spectrum 100 FTIR spectrophotometer) of drug-loaded PVA films was carried out (at 256 scans, 8 cm^−1^ resolution) to investigate the presence of functional groups and types of interaction between the drug and polymeric components.

## 3. Results and Discussion

### 3.1. Degradation Studies

**Figure 1:**
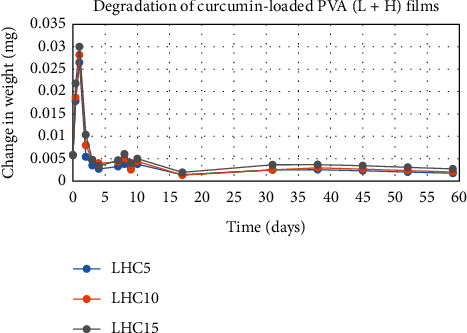
Degradation of curcumin-loaded PVA (L + H) films.

**Figure 2:**
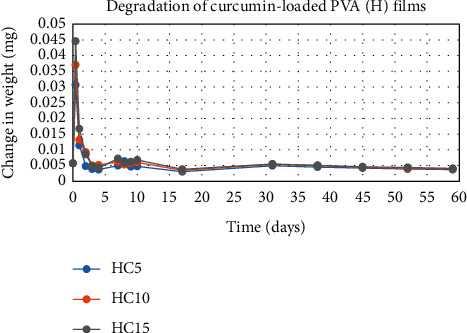
Degradation of curcumin-loaded PVA (H) films.

**Figure 3:**
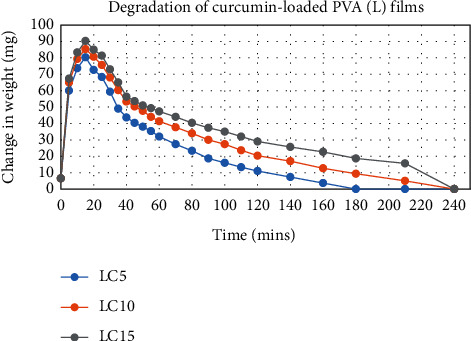
Degradation of curcumin-loaded PVA (L) films.

**Figure 4:**
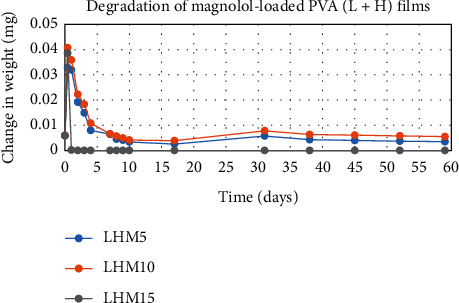
Degradation of magnolol-loaded PVA (L + H) films.

**Figure 5:**
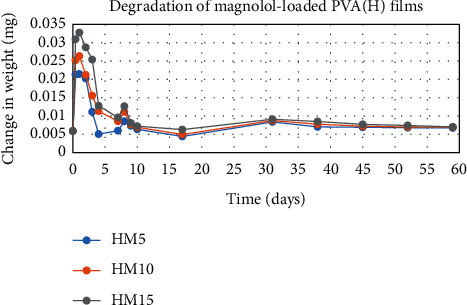
Degradation of magnolol-loaded PVA (H) films.

**Figure 6:**
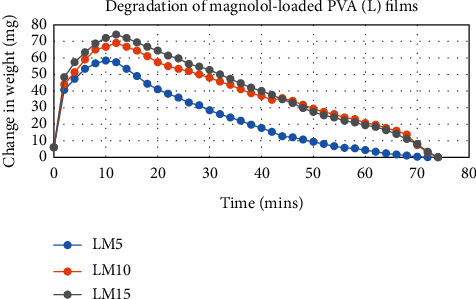
Degradation of magnolol-loaded PVA (L) films.

**Figure 7:**
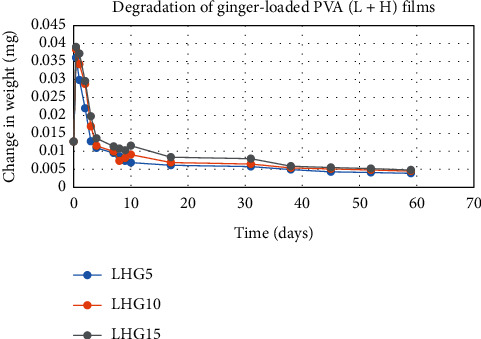
Degradation of ginger-loaded PVA (L + H) films.

**Figure 8:**
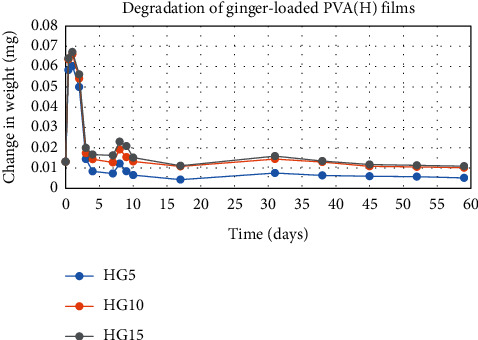
Degradation of ginger-loaded PVA (H) films.

**Figure 9:**
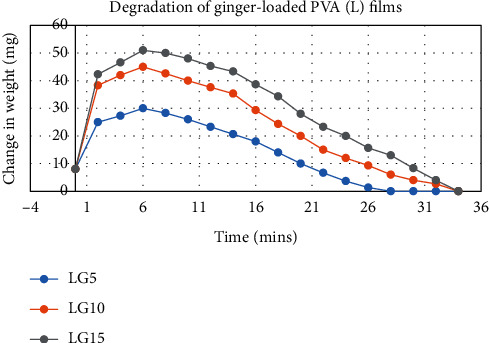
Degradation of ginger-loaded PVA (L) films.

The degradation profile of composites was evaluated by recording weight loss at predetermined time intervals (Figures 1–9). The degradation profile of all drug-loaded PVA (H) and PVA (L + H) films was divided into two stages; during first 48 hours, a sudden increase in the weight of the films was observed (Figures 1, 2, 4, 5, 7, and 8) because of the absorption of the buffer solution by PVA followed by a decrease in weight, which continued for more than 60 days (the study was conducted for 60 days). This behavior of PVA was due to the reason that PVA, when exposed to aqueous media, absorbs the liquid and swells, resulting in an increase in weight; later, it becomes solvated and starts losing mass [28, 29]. Although all the PVA (L) compositions were degraded within few minutes, for example, ginger-loaded films took only 35 minutes to degrade completely (100% degradation) (Figure 9), the curcumin-loaded polymeric matrix took approximately 235 minutes to degrade (Figure 3) and 70 minutes were taken by magnolol-loaded films to degrade completely (Figure 6). Thus, all the drug-loaded films of PVA (H) and PVA (L + H) took longer time to degrade (more than 60 days) (Figures 1, 2, 4, 5, 7 and 8) as compared with the polymeric matrix with PVA (L) (degraded within few minutes) (Figure 3, 6 and 9).

It was established from the degradation results that with the increase in amount of drug content, the degradation rate was decreased, that is, degradation of ginger (PVA (L + H)) was 69% when its concentration was 5%; however, when the ginger concentration was increased to 15%, the degradation rate was decreased to 62% (Figure 7). Thus, it can be concluded that with the increase in drug content, the degradation rate of PVA decreases and it can be explained in terms of PVA interactions with the drug. As the drug concentration increases, the interaction of the drug with the polymer also increases, which results in increased bonding between the drug and polymer. This increased bonding results in less exposure of PVA hydroxyl groups to water, thus subsequently decreasing the rate of degradation [30].

Another observation that was made from degradation graphs is that the degradation rate was higher in the case of PVA (L + H) as compared with the polymeric matrix with PVA (H) (Figures 1, 2, 4, 5, 7 and 8); for example, degradation was 93% for LHG5 and 61% for HG5 (Figures 7 and 8). The intermolecular interaction in PVA (H) films is stronger when measured against PVA (L + H) films. The PVA (L) in blend caused the films to become easily solvated, resulting in the loss of weight and thus increasing the degradation rate unlikely to PVA (H) because PVA (L) tends to uptake water quickly and subsequently the OH groups of PVA dissolves in water, exposing the corresponding functional groups to a dissolution medium, resulting in faster degradation rates. While in the case of PVA (H) due to lengthening of polymeric chains and its intermolecular interaction, the uptake of water is slow, leading to slow degradation of the polymer.

### 3.2. Drug Release Studies

**Figure 10:**
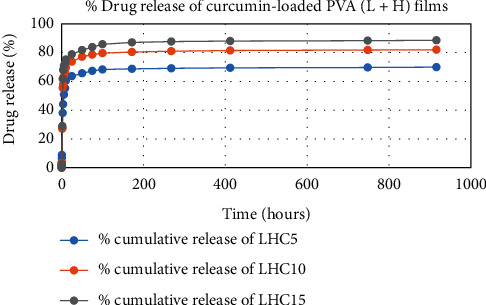
Drug release % of curcumin-loaded PVA (H + L) films.

**Figure 11:**
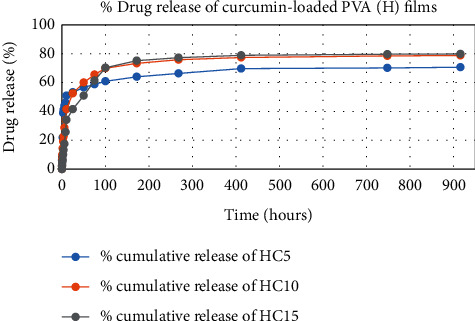
Drug release % of curcumin-loaded PVA (H) films.

**Figure 12:**
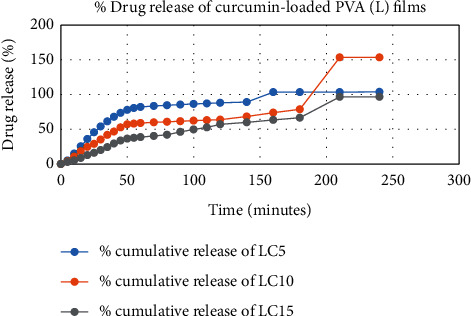
Drug release % of curcumin-loaded PVA (L) films.

**Figure 13:**
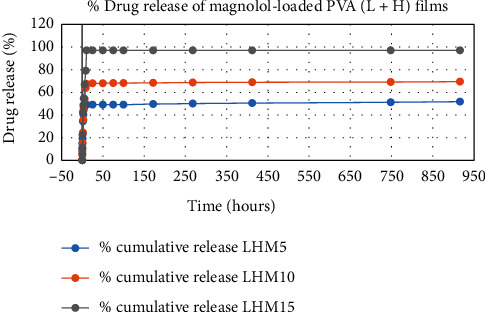
Drug release % of magnolol-loaded PVA (H + L) films.

**Figure 14:**
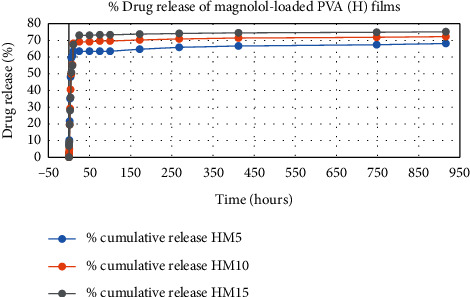
% Drug release % of magnolol-loaded PVA (H) films.

**Figure 15:**
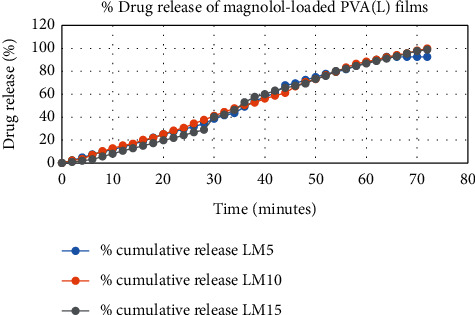
Drug release of magnolol-loaded PVA (L) films.

**Figure 16:**
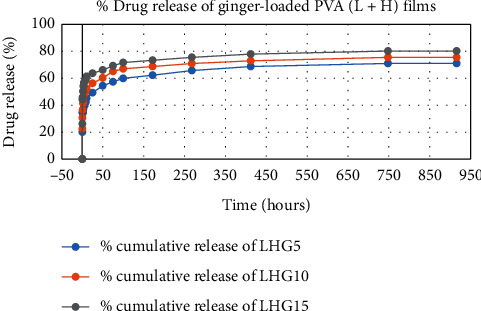
Drug release % of ginger-loaded PVA (H + L) films.

**Figure 17:**
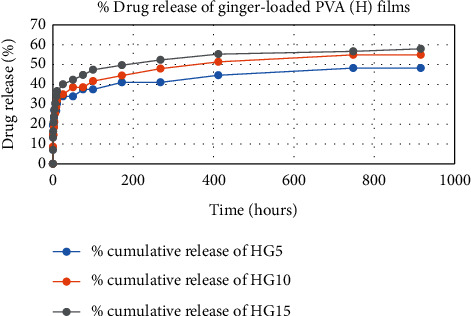
Drug release % of ginger-loaded PVA (H) films.

**Figure 18:**
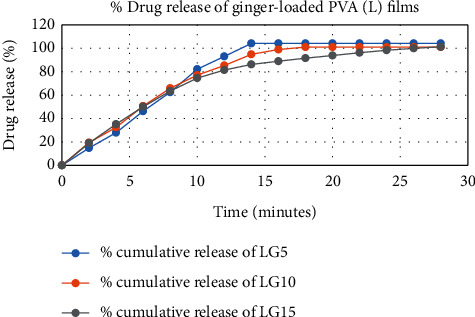
Drug release % of ginger-loaded PVA (L) films.

The results of in vitro elution of drugs from different types of drug-loaded films are shown in Figures 10–18. Generally, drug release from a polymer-based matrix takes place with an initial burst release [31]. As shown in corresponding figures, all the drug-loaded films of PVA (H) and PVA (L + H) exhibited an initial burst release during first 48 hours followed by a nearly linear sustained-release profile over a period of 38 days. The initial burst release occurred because PVA uptakes water and swells, and this swelling allowed drug to diffuse out from polymer matrix into the release medium. Later, a slow drug release happened because of slow degradation of the polymer matrix. Moreover, replacing the releasing medium every day established a significant concentration gradient of drug between the drug-eluting film and the release medium and thus led to linear constant drug diffusion [32]. On the other hand, because PVA (L) degrades completely within first few minutes, it released all the drug content into the medium within that duration following the similar behavior of the initial burst release followed by slow release as observed in drug-loaded films of PVA (H) and PVA (L + H).

In theory, a sustained release of antirestenotic drugs for at least three weeks is required to prevent smooth muscle cell migration and proliferation [33]. Based on the release profile shown above, the average release percentages of curcumin, ginger, and magnolol for low-dose films of PVA (L + H) were 0.69%, 82%, and 88%, for moderate-dose films were 70%, 75%, and 80%, and for high-dose films were 51%, 69%, and 100%, respectively, for 38 days of release. Thus, based on these release rates, it can be concluded that the release duration was ∼38 days for curcumin, ∼31 days for ginger, and ∼35 days for magnolol for all the compositions of LH and H (all entirely satisfactory for a releasing requirement of three weeks).

### 3.3. Hemolytic Activity

**Figure 19:**
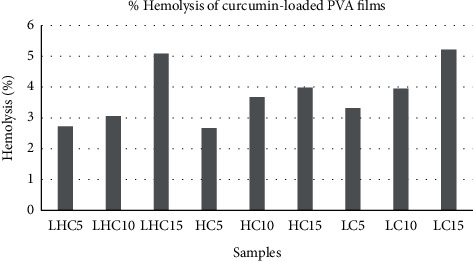
Hemolysis release % of curcumin-loaded PVA films.

**Figure 20:**
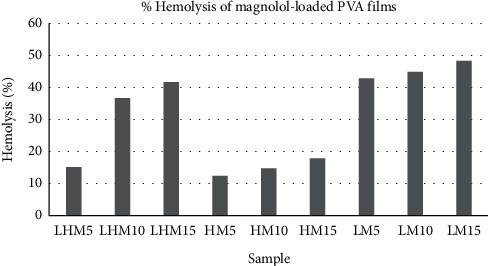
Hemolysis release % of magnolol-loaded PVA films.

**Figure 21:**
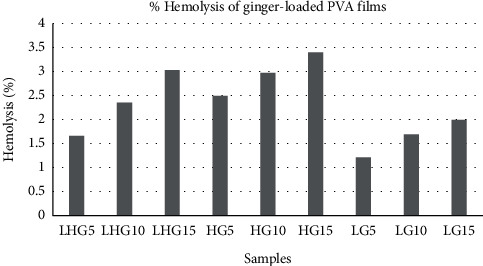
Hemolysis release % of ginger-loaded PVA films.

The results of hemolytic assay are the direct indicator of hemocompatibility of the drug-loaded PVA films. Thus, in vitro hemolytic activity of three natural components on human red blood cells was performed and is presented in Figures 19–21. 100% hemolytic activity was gained through 0.5% Triton X-100 (positive control), while 0% hemolysis was obtained using PBS (negative control).

Through the results of percentage hemolysis of drug-loaded polymeric films, it was observed that LHM5 showed 15% hemolysis, which was increased to 36% in the case of LHM10 and 41% for LHM15 (Figure 20). However, in the case of curcumin, the increase was from 2.7% to 5.08% (Figure 19) when the concentration was shifted to 15% from 5%. Moreover, the hemolysis graph of ginger showed 1.6% hemolysis caused by ginger when the concentration was 5%, which was increased to 2.3% and 3.02% in the case of polymeric films with 10% and 15% ginger concentrations (Figure 21). This phenomenon can be explained in terms of amount of drug released in the extracellular environment. The loaded drug is present on the surface of the polymeric film; consequently, the drug was released in the environment to interact with red blood cells. Moreover, increasing the drug amount subsequently increases the release of the drug as shown in drug release graphs, thus making the drug available to cause the hemolytic effect on RBCs. The fact that the increase in drug content in polymeric films results in the increase in release of drug in the release medium, which was also established by Pan et al. [34].

In addition, it was also observed that the molecular weight of PVA influences the effect of drugs on human erythrocytes. The PVA (L) has positive impact on the activity of curcumin and magnolol (activity was 3.3% for LM5, which was decreased to 2.6% and 2.7% for HM5 and LHM5, respectively) as compared with PVA (H) films and the matrix of PVA (L + H), whereas the positive effect on the activity of ginger was caused by PVA (H) instead of PVA (L), such as the hemolysis percentage was 2.4% with HG5, which was decreased to 1.6 % and 1.2 % with LHG5 and LG5, respectively. Thus, it can be concluded that the molecular weight of polymer affects the activity of drug by controlling the release of the drug from the polymeric matrix. The potential reason may be that PVA (L) degrades within few minutes, dispensing the maximum amount of drug in the release medium to act on human erythrocytes [35] Furthermore, effects of the molecular weight of polymer on the drug release has been demonstrated by Mittal et al. [36], and they concluded that increasing the molecular weight increases the lipophilicity, which decreases the degradation rate of the polymer, resulting in decreased release of drug from the polymer matrix [36]. However, in polymeric film (PVA (H)), ginger is present on the surface with weak physical interactions with polymer, released in the aqueous environment liberating hemoglobin in extra cellular environment, thus causing hemolysis of RBCs [35].

A number of research groups have reported the hemolytic activity of the drugs used in the present study. Jawad et al. have reported the hemolytic activity of magnolol up to 11.9% when 64 *μ*g/mL of drug concentration was used [37]. On the other hand, Kumar et al. in 2011 and 2012 investigated the blood compatibility of silver and gold nanoparticles incorporated with ginger extract. They found negligible hemolytic effect of nanoparticles and thus concluded that ginger extract-based nanoparticles can be used for drug delivery applications as they are safe when they come into contact with blood [38,39]. According to the ASTM F756 standard, depending on hemolytic activity, materials can be classified into three different categories: Materials resulting in over 5% hemolysis are classified as hemolytic, between 5% and 2% as slightly hemolytic, and below 2% as nonhemolytic [40]. It was established after analyzing the hemolytic results of drug-loaded polymeric films that polymer matrices such as LHG5, LG5, LG10, and LG15 are nonhemolytic according to the ASTM standard, whereas all the polymeric films of curcumin and ginger are slightly hemolytic, and magnolol-loaded PVA films are hemolytic. Therefore, according to the ASTM standard, films classified as nonhemolytic are potential candidates that are to be used as drug-coating materials for drug-eluting stent application.

### 3.4. Scanning Electron Microscopy (SEM)

**Figure 22:**
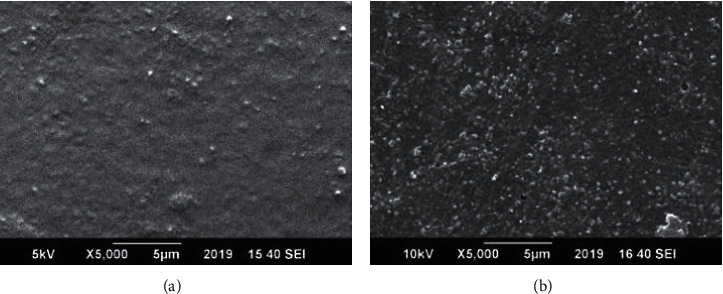
SEM images of (a) PVA (L + H) and (b) 5% ginger-loaded PVA (L + H) film.

The surface morphology of ginger-loaded PVA films, selected on the basis of its good hemocompatibility, was assessed by SEM, which has been demonstrated in Figure 22. The SEM images showed the aggregates of ginger dispersed on the surface of films, which contributed to the increase in the roughness of the film surface. Furthermore, the presence of aggregates on the surface of PVA was the main reason of the initial burst release of ginger from the polymeric films during drug release studies and the cause of weak physical interactions with the polymer. Similar results have been reported by Pereira et al. while studying the properties of alginate-based aloe vera films [41].

### 3.5. Fourier Transform Infrared (FTIR) Analysis

**Figure 23:**
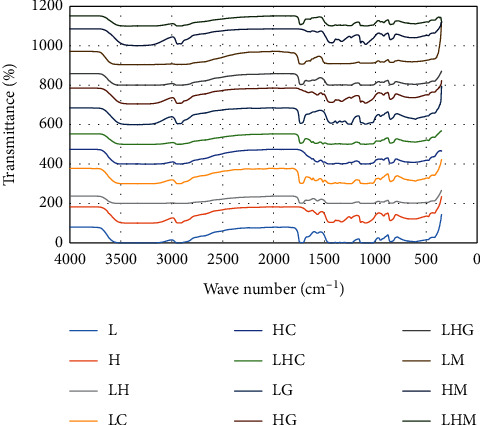
FTIR spectra of drug-loaded polymeric films.

FTIR spectra for PVA/curcumin, PVA/magnolol, and PVA/ginger polymeric films are shown in Figure 23. The spectrum of PVA showed the characteristic peaks of OH at 3319 cm^−1^ and stretching vibrations of CH2/CH groups at 2941/2912 cm^−1^ and C–O at 1734 cm^−1^. The deformation bands of CH2/CH and C–O stretching vibrations were observed at 1435/1375 cm^−1^, 1096 cm^−1^, and 1258 cm^−1^, respectively [42].

For curcumin, the bands observed at 3085–3552 cm^−1^, 1588 cm^−1^, 1512 cm^−1^, 1265 cm^−1^, and 1143 cm^−1^ are attributed to the phenolic O–H stretching, stretching vibrations of the benzene ring, C–C vibrations, aromatic C–O stretching, and C–O–C stretching modes, respectively [42].

In FTIR spectra of magnolol, the intense absorption band attributed to the hydroxyl stretching vibration occurs at 3138 cm^−1^. The presence of allyl C=C stretching vibration is determined by the absorption band at 1637 cm^−1^, while C=C aromatic stretching is characterized by the 1494 cm^−1^ absorption band. The broad peak in the range of 3340–2663 cm^−1^ includes the stretching vibration of aliphatic C–H, C–O, and Ar–H bending vibrations [43].

In the case of ginger, the presence of bands with strong to medium intensities were reported at 2922 cm^−1^ and 2870 cm^−1^, which confirmed the COOH groups. Other strong-to-medium intensity bands at 2731 cm^−1^ also described the presence of aldehyde groups, and the peak at 1712 cm^−1^ indicated the presence of carbonyl compounds. Some other bands reported intensities at 1636 cm^−1^ and 1609 cm^−1^, 1514 cm^−1^ and 1459 cm^−1^, 1375 cm^−1^, 1272 cm^−1^, 1159 cm^−1^, 1037 cm^−1^, and 618 cm^−1^ attributed towards the presence of alkene (CH2CH2), CC aromatic, alkene, methyl CH3, ether (ROR), alcohol primer, and phenol, respectively [44, 45].

The spectrum of the 5 wt% curcumin-loaded PVA films is shown in Figure 23. The FTIR pattern of curcumin and curcumin-loaded films was distinguished from that of PVA membrane because of the peaks at 3447 cm^−1^, 1585 cm^−1^, 1265 cm^−1^, and 1096 cm^−1^, which correspond to phenolic OH stretching, stretching vibrations of benzene ring, aromatic C=O vibration, and C–O–C vibration modes of curcumin, respectively. This indicated few interactions between the drug and polymer, suggesting that the drug is in dispersed form in the polymeric matrix.

The interactions between ginger and PVA are shown by the FTIR spectrum in Figure 23. The spectrum comprises the band ranges of pure PVA, ginger, and blend of ginger and PVA. The peaks observed at 2935 cm^−1^ of CH2 stretching, 1732 cm^−1^ corresponding to C=O vibrations, 1651 cm^−1^ amide stretching, and 1100 cm^−1^ ester group showed the presence of ginger in drug/polymer films, indicating physical bonding between the drug and polymer.

In Figure 23 the FTIR band range for magnolol was shown. The peaks at 1635 cm^−1^, 1455 cm^−1^, 1118 cm^−1^, and 916 cm^−1^ indicated the characteristic peaks—alkyl stretching vibration of C=C, C=C aromatic stretching, ester bond, and C–O stretching of aliphatic ether group, respectively—of magnolol, demonstrating the weak bonding among magnolol and the polymer and scattering of magnolol in the polymeric film matrix. The IR spectra of all the compositions confirmed the presence of respective drugs in the polymer network.

## 4. Conclusion

In the current study, three different plant extracts (curcumin, ginger, and magnolol) loaded on biodegradable polymeric films were investigated through in vitro drug release and degradation profile in addition to hemolysis assay and were proposed as potential coating materials for drug-eluting stents for the treatment of cardiovascular diseases. The outcomes of the drug release study showed an initial burst release with all three drug-loaded polymeric films followed by a sustain release. Subsequently, the results of hemolysis assay carried out in this study concluded that ginger with 5% concentration gave maximum hemocompatibility, while magnolol with all its concentration exhibited hemotoxicity towards human red blood cells. Furthermore, all three concentrations of curcumin gave percentage hemolysis that lies in the category of slightly hemolytic according to the ASTM F756 standard. Thus, ginger-loaded polymeric films are more hemocompatible, and after further blood compatibility tests such as activated partial thromboplastin time (APTT) and cytotoxicity tests, these films can be used as stent-coating materials. After conducting further biocompatibility tests on the films prepared in the current study, ginger-loaded PVA solution composition can be a future candidate to be used as a coating material for developing drug-eluting stents.

## Figures and Tables

**Table 1:** Composition of curcumin-based PVA films.

S. no.	Sample ID	PVA (L)	PVA (H)	PVA (L + H)	Curcumin(%)
1	LC5	L	—	—	5
2	LC10	L	—	—	10
3	LC15	L	—	—	15
4	HC5	—	H	—	5
5	HC10	—	H	—	10
6	HC15	—	H	—	15
7	LHC5	—	—	L + H	5
8	LHC10	—	—	L + H	10
9	LHC15	—	—	L + H	15

**Table 2:** Composition of magnolol-based PVA films.

S. no.	Sample ID	PVA (L)	PVA (H)	PVA (L + H)	Magnolol(%)
1	LM5	L	—	—	5
2	LM10	L	—	—	10
3	LM15	L	—	—	15
4	HM5	—	H	—	5
5	HM10	—	H	—	10
6	HM15	—	H	—	15
7	LHM5	—	—	L + H	5
8	LHM10	—	—	L + H	10
9	LHM15	—	—	L + H	15

**Table 3:** Composition of ginger-based PVA films.

S. no.	Sample ID	PVA (L)	PVA (H)	PVA (L + H)	Ginger (%)
1	LG5	L	—	—	5
2	LG10	L	—	—	10
3	LG15	L	—	—	15
4	HG5	—	H	—	5
5	HG10	—	H	—	10
6	HG15	—	H	—	15
7	LHG5	—	—	L + H	5
8	LHG10	—	—	L + H	10
9	LHG15	—	—	L + H	15

## Data Availability

Supplementary data are available from the corresponding author upon request.
